# One Century of Forest Monitoring Data in Switzerland Reveals Species- and Site-Specific Trends of Climate-Induced Tree Mortality

**DOI:** 10.3389/fpls.2019.00307

**Published:** 2019-03-22

**Authors:** Sophia Etzold, Kasia Ziemińska, Brigitte Rohner, Alessandra Bottero, Arun K. Bose, Nadine K. Ruehr, Andreas Zingg, Andreas Rigling

**Affiliations:** ^1^Swiss Federal Institute for Forest, Snow and Landscape Research WSL, Birmensdorf, Switzerland; ^2^SwissForestLab, Birmensdorf, Switzerland; ^3^Institute of Meteorology and Climate Research – Atmospheric Environmental Research, Karlsruhe Institute of Technology, Garmisch-Partenkirchen, Germany; ^4^Institute of Terrestrial Ecosystems, ETH Zurich, Zurich, Switzerland

**Keywords:** drought, competition, stand basal area, climate change, tree size, mortality

## Abstract

Climate-induced tree mortality became a global phenomenon during the last century and it is expected to increase in many regions in the future along with a further increase in the frequency of drought and heat events. However, tree mortality at the ecosystem level remains challenging to quantify since long-term, tree-individual, reliable observations are scarce. Here, we present a unique data set of monitoring records from 276 permanent plots located in 95 forest stands across Switzerland, which include five major European tree species (Norway spruce, Scots pine, silver fir, European beech, and sessile and common oak) and cover a time span of over one century (1898–2013), with inventory periods of 5–10 years. The long-term average annual mortality rate of the investigated forest stands was 1.5%. In general, species-specific annual mortality rates did not consistently increase over the last decades, except for Scots pine forests at lower altitudes, which exhibited a clear increase of mortality since the 1960s. Temporal trends of tree mortality varied also depending on diameter at breast height (DBH), with large trees generally experiencing an increase in mortality, while mortality of small trees tended to decrease. Normalized mortality rates were remarkably similar between species and a modest, but a consistent and steady increasing trend was apparent throughout the study period. Mixed effects models revealed that gradually changing stand parameters (stand basal area and stand age) had the strongest impact on mortality rates, modulated by climate, which had increasing importance during the last decades. Hereby, recent climatic changes had highly variable effects on tree mortality rates, depending on the species in combination with abiotic and biotic stand and site conditions. This suggests that forest species composition and species ranges may change under future climate conditions. Our data set highlights the complexity of forest dynamical processes such as long-term, gradual changes of forest structure, demography and species composition, which together with climate determine mortality rates.

## Introduction

Forest ecosystems play a crucial role in maintaining the balance of land-atmosphere cycles, sequestering carbon, fostering biodiversity and providing esthetic value to humanity (Bonan, 2008; Pan et al., 2011; Millar and Stephenson, 2015). Hence, understanding forest mortality is a pressing task of our times in order to predict and regulate the future development of forests. Although forest mortality is a natural process within forest stand dynamics (Franklin et al., 1987), mortality rates have been reported to increase in recent years due to climatic changes (Allen et al., 2010; McDowell et al., 2018), and are predicted to increase further in the future (Steinkamp and Hickler, 2015).

Drivers of tree mortality are diverse and intertwined with one another. The decline-disease theory is a conceptual framework relating tree mortality to a sequence of abiotic and biotic factors, which can be of predisposing, inciting or contributing nature (Manion, 1981; Houston, 1984). Predisposing factors (e.g., age or air pollutants) devitalize a tree over a long time period. Inciting factors, such as drought or insect defoliation, are short-term events that considerably reduce tree vigor. Finally, the occurrence of contributing factors (e.g., additional drought events, fungi) determines the fate of the weakened tree. Contributing factors can act in a long term, but can also cause a fast die-off when a certain threshold is reached (e.g., bark beetle infestations). Forest stand characteristics play an important role in forest mortality. For example, higher stand basal area and subsequent competition have been shown to increase mortality rates (Bradford and Bell, 2017; Young et al., 2017). Mortality risk also depends on tree or stand age (van Mantgem et al., 2009; Cailleret et al., 2017; Neumann et al., 2017) with usually a U-shaped curve of mortality probability in relation to age and/or tree size (Coomes and Allen, 2007; Lines et al., 2010). In recent years, changing climatic conditions, such as increasing drought intensity and frequency as well as rising temperatures, have been found to strongly influence forest mortality rates (van Mantgem et al., 2009; Allen et al., 2010; Anderegg et al., 2013). Disturbances that are often a consequence of changing climate, such as more numerous and severe fires (Brando et al., 2014; Stephens et al., 2018) or damaging insect outbreaks associated with storms and droughts (Weed et al., 2013; Kolb et al., 2016; Wood et al., 2018) have also been linked to increased forest mortality. Given this diversity of factors, mortality rates vary depending on the geographical location, its climate and consequent vegetation structure. Nevertheless, an increase in forest mortality due to climatic changes has been observed across continents, under different climatic conditions and across diverse forest types, from gymnosperm to angiosperm dominated ones, and from tropical to boreal regions (Allen et al., 2010; Anderegg et al., 2015; McDowell et al., 2018).

Drought-induced tree mortality can develop after substantial long periods of suboptimal water supply. The underlying physiological mechanisms include substantial damages to the tree hydraulic system through air entering the water conducting tissues of trees (Tyree and Sperry, 1989) under high xylem tensions induced by low soil moisture and high evaporative demand during drought. If these so called embolisms are substantial, then trees are not able to recover (Hartmann et al., 2018) following drought release and may subsequently die (Anderegg et al., 2016; Adam et al., 2017). Trees have developed multiple physiological strategies, at root, stem and leaf level, which allow them to cope with drought stress and to avoid hydraulic failure to a certain degree. For example, they can grow deep roots for better access to water, build xylem that is robust to embolism and/or control water loss via stomatal closure or leaf shedding (Martin-StPaul et al., 2017; Choat et al., 2018).

Growing recognition of the impacts of extreme droughts on forest ecosystems in Europe has spurred scientific attention on how major tree species including Scots pine (*Pinus sylvestris* L.), Norway spruce [*Picea abies* (L.) H. Karst], silver fir (*Abies alba* Mill.), European beech (*Fagus sylvatica* L.) and oak [*Quercus petraea* (Matt.) Liebl., *Quercus robur* L.] will cope with the expected changes in climate. These species follow different physiological strategies to cope with drought stress and exhibit different levels of drought tolerance (Breda et al., 2006). Among these species, Scots pine usually occurs on the driest sites used for commercial forestry in Central Europe. Compared to Norway spruce and silver fir, Scots pine is considered to be more drought tolerant. This is presumably due to its early stomatal closure to avoid xylem embolism under mild-moderate drought (Martinez-Vilalta et al., 2004) in combination with a deep rooting system (Richardson, 2000). Despite these strategies, Scots pine may not be well adapted to the combined effects of drought and heat (Dobbertin et al., 2005; Giuggiola et al., 2010) or to several consecutive drought events (Bigler et al., 2006) and is further vulnerable to diverse drought-related pests and diseases (Wermelinger et al., 2008). Norway spruce has repeatedly been described as particularly susceptible to drought (Zang et al., 2012, 2014; Pretzsch et al., 2013), which is likely due to its shallow root system and its vulnerability to drought-related insect outbreaks. There is an agreement that Norway spruce is already negatively affected by the impacts of climate change, which will be more pronounced in the future (Hanewinkel et al., 2013; Zang et al., 2014). Silver fir, however, has been shown to be more resilient to drought stress and associated phenomena like bark beetle outbreak or storm damages (Zang et al., 2014; George et al., 2015; Vitali et al., 2017). European beech is another drought sensitive species (Leuschner et al., 2001; Gessler et al., 2007; Charru et al., 2010). Several studies show a strong decline in radial growth of beech trees in response to decreasing water availability particularly for the Mediterranean region (Jump et al., 2006; Piovesan et al., 2008). However, beech has also shown a high physiological plasticity and adaptability to changing growing conditions (Vitasse et al., 2014; Cocozza et al., 2016; Stojnic et al., 2018), and the acclimation of beech to future climatic changes is highly uncertain. Oaks are generally considered to be well adapted to drought, due to their high resistance to xylem embolism (Robert et al., 2017; Lobo et al., 2018), their high capacity to withdraw water from the soil also under dry conditions by deep root systems and their ability to maintain water potential gradients along the soil-stem-crown continuum (Zweifel et al., 2007), as well as their xeromorphic leaf structure. Moreover, they are able to rapidly resume assimilation after periods of water deficiency (Kubiske and Abrams, 1993; Kuster et al., 2013).

Across Europe, a considerable number of studies found increasing mortality rates due to drought and heatwave events in Spain (Peñuelas et al., 2001; Martínez-Vilalta and Piñol, 2002), France (Bréda et al., 2006; Vennetier et al., 2007), Switzerland (Bigler et al., 2006), Poland (Siwecki and Ufnalski, 1998), Norway (Solberg et al., 2004), and Russia (Kauhanen et al., 2008; Ogibin and Demidova, 2009). However, opposite trends in mortality rates have also been reported. When comparing the period of 1900–1960 with 1960–2000, mortality rates of Norway spruce in Germany did not change, while for beech, mortality rates decreased by 17% (Pretzsch et al., 2014b). Similarly, annual mortality rates of four angiosperm species in Sweden were also found to be smaller in recent decades (1988–2013) than earlier (1912–1988, Hytteborn et al., 2017). Our current understanding on forest mortality trends is strongly influenced by the time span of available data. A long-term perspective is necessary to obtain a comprehensive picture of forest dynamics, especially when dealing with long-living organisms, such as trees.

It is also important to make a distinction between mortality rates of individual species and mortality rates of entire forest ecosystems. Studies investigating physiological mechanisms driving mortality usually focus on species-specific mortality, and often cover a relatively short time span, limited sample size and geographical range (e.g., Chao et al., 2008; Mitchell et al., 2013). In contrast, ecosystem-scale mortality rates are typically assessed across multi-year or multi-decadal time span, covering a large sample size and geographical range (e.g., Williams et al., 2013; Neumann et al., 2017; Huelsmann et al., 2018). The two perspectives complement each other and are essential for improving our current understanding and future predictions of tree and forest mortality across the globe.

The majority of ecosystem-scale studies assess mortality rates across one or two decades (e.g., Monserud and Sterba, 1999; Bradford and Bell, 2017; Pillet et al., 2018; Rogers et al., 2018) or after a specific disturbance event (e.g., Breshears et al., 2005; Michaelian et al., 2011; Brodrick and Asner, 2017; Reed et al., 2018). However, long-term mortality trends can add a unique perspective and significantly improve our understanding of forest dynamics. Long-term monitoring requires extensive effort, resources and commitment; hence, the data are extremely scarce. We are aware of only three studies reporting tree mortality rates of seven European species and spanning a time frame of at least 100 years (Pretzsch et al., 2014a,b; Hytteborn et al., 2017). An additional challenge is to assess mortality across broad climatic gradients and across a more diverse suite of species. For example, a recent geographically comprehensive study encompassed entire Europe, but included only two species and covered one decade (Neumann et al., 2017).

In this study, we address these knowledge gaps by compiling a dataset spanning *ca*. 120 years of inventory data for five dominant European tree species in Switzerland: Scots pine, Norway spruce, silver fir, European beech, sessile and common oak. Despite the fact that our study is geographically restricted to Switzerland, the large heterogeneity of the Swiss landscape enables an assessment of forest mortality under a wide range of environmental conditions. For example, the 276 sampled plots spanned a wide altitudinal (∼1800 m) and precipitation gradient (∼800 mm during growth season). We combined long-term inventory data of forest mortality, stand characteristics such as basal area and mean stand diameter at breast height (used as proxy for stand age), and high-resolution climate data to: (i) assess annual tree mortality rates and temporal trends of five dominant tree species across Switzerland over the last century, and (ii) identify the main drivers of mortality, out of a set of 12 predictors, including climate, stand characteristics and topography. We hereby focused on the impact of summer drought conditions on mortality, being aware that also other climatic factors, such as winter conditions, might have an impact on forest mortality at certain sites. We hypothesized that drought-induced mortality is species-specific with high drought-induced mortality rates in spruce and beech and lowest mortality rates in more drought-resistant oak and pine. Within species we expected different responses to drought along environmental gradients, due to different limiting factors that depend on site conditions (e.g., temperature *vs*. water availability). For instance, pine or spruce growing at dry (lowland) sites and showing already climate related declines should exhibit higher drought induced mortality rates compared to wet or high-altitude sites. On the other hand, it has been shown that, e.g., beech trees that are adapted to a generally lower water availability are more resistant to occurring droughts compared to beech trees growing under generally good water supply (Grossiord et al., 2014; Kunz et al., 2018). Therefore, we would expect a higher drought-induced mortality on wet sites compared to drier sites for species with a high adaptive capacity, e.g., beech and oak.

## Materials and Methods

### Forest Inventory Data

The study includes data from three long-term data sources of Swiss forest growth monitoring networks (Supplementary Table S1): Swiss Experimental Forest Management plots network (EFM), Swiss Long-Term Forest Ecosystem monitoring network (LWF), and the Swiss Nature Reserves Network (SNR). Within all three networks, the diameter at breast height (DBH, 1.3 m height) and the status (dead/alive) of all marked trees larger than a network-specific DBH threshold were recorded during repeated inventory campaigns (Supplementary Table S1). For consistency, only trees with DBH > 5 cm were included in this study.

The EFM plots were established between 1887 and the early 1900s, and currently comprise *ca*. 390 yield plots across Switzerland in order to study the development of forests in a changing environment under the influence of forest management (cf., Schütz and Zingg, 2010). Inventory intervals ranged from 3 to 22 years (mean 7 years). The LWF plots were established in 1995 and are part of the intensively monitored Level II plot network of the International Co-operative Programme on Assessment and Monitoring of Air Pollution Effects on Forests (ICP Forests) in Europe (Ferretti et al., 2010). The LWF plots are assessed every 3–6 years (mean 5 years) since 1995 according to a harmonized sampling protocol (Dobbertin and Neumann, 2010; Hug et al., 2011). The SNR network currently consists of 49 unmanaged forest reserves throughout Switzerland (Brang et al., 2006, 2011; Wunder et al., 2007). Repeated inventory campaigns of core plots within the nature reserves have been conducted every 5–20 years (mean 12 years) since the late 1940s. It needs to be noted that the permanent plots of the three monitoring networks were assessed at different intervals (3–22 years, on average 9 years), for different inventory years and time periods, and that the number of plots increased with time.

### Study Sites

**FIGURE 1:**
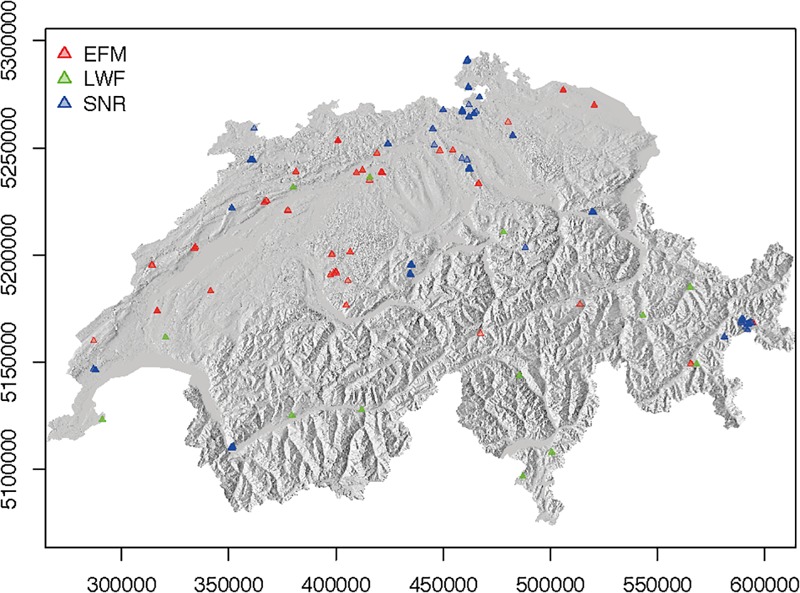
Location of plots from EFM (red symbols), LWF (green symbols) and SNR (blue symbols) networks included in this study. In a number of locations, several plots are located in close proximity to each other. See Supplementary Material for maps showing locations of each species separately (Supplementary Figure S1).

A total of 276 permanent plots within 95 forest sites (Supplementary Table S1 and Figure 1) were selected according to the following criteria: (1) at least two inventories per plot were available; (2) the plots were not affected – as far as documented – by major disturbances or management interventions; for the EFM, only plots with natural thinning (A grade according to IUFRO rules, Versuchsanstalten, 1902) or plots with low management intensity, where suppressed, fallen or dying stems are removed from below but without impact on crown layer (B grade), were included, so that the measured mortality rates should reflect as far as possible the natural mortality rates; (3) plots consisted of at least 20% of the basal area of one of the following species: Scots pine (*Pinus sylvestris* L.), Norway spruce [*Picea abies* (L.) H. Karst], silver fir (*Abies alba* Mill.), European beech (*Fagus sylvatica* L.) or oak [*Quercus petraea* (Matt.) Liebl., *Quercus robur* L.]. The two oak species were treated as one entity and are referred throughout the text as ‘oak’, due to their morphological similarities and tendency to hybridize, leading to their disputed taxonomical identity (Kissling, 1980; Aas, 1998; Muir et al., 2000).

The plots within the SNR and the EFM forest sites were considered as separate plots, despite their geographical proximity (on average 100 m apart, ranging from 3 to 423 m), as their stand structure and abiotic characteristics were very heterogeneous (cf. Brang et al., 2011). The individual plots covered an area of 0.02–3.47 ha (mean 0.5 ha). Out of the total of 276 plots, 175 were pure stands (basal area of a given species > 70%) and 101 mixed stands. Among the pure stands, 47 were dominated by pine, 23 by spruce, 10 by fir, 42 by beech, 50 by oak, and 3 by other species (for these plots, species with remaining 20–30% basal area were included in analyses). According to annual changes in tree numbers and basal area as described in Brang et al. (2011), 40 stands were classified to the juvenile forest phase, 17 stands to the young forest phase, 178 to the optimal phase and 41 to the old growth phase. The mean stand age of the 100 forest stands with age information was 130 years (ranging from 45 to 360 years). In total, the data series used in this study consisted of 2–17 inventories per plot (on average 9) from 1898 to 2013. Average data series length was 45 years and the longest series comprised 113 years from 1898 to 2011. In total, data of 247,529 trees were included.

### Climate Data

All site-specific climate data (temperature, precipitation, drought stress indicators) for the time period from 1901 to 2012 were obtained with a daily resolution from Remund et al. (2016). Climate data from 1901 to 1930 were interpolated from CRU grid data “CRU TS Version 1.2” (Mitchell et al., 2004) based on the change factor method, and since 1931 from observational plots (MeteoSchweiz) based on the Shepard’s Gravity interpolation method (Zelenka et al., 1992).

For all study plots within one forest site identical climate data were used. All climate variables were calculated for the growth period from May to September. The minimal site water balance (SWB_min_) during the growth period was used as drought indicator as it was found to be one of the best predictors of mortality in a comparison of various multivariate models for beech and spruce in Switzerland (Braun, 2016). In addition, SWB_min_ resulted in the best agreement with soil water content measured in 10–20 cm soil depth for a subset of LWF sites used in this study, relative to other drought indices (*R* = 0.48, data not shown). SWB_min_ was calculated according to Grier and Running (1977) as sum of the daily differences between precipitation sum and potential evapotranspiration with field capacity as starting value. The detailed methodology is described here: http://www.wsl.ch/staff/niklaus.zimmermann/programs/amls/swb.aml. Lower SWB_min_, denotes higher drought stress. In this study, the minimal value for the growth season was applied.

### Ecoregions

Within each species, plots were categorized into two distinct groups, referred to as “ecoregions” (Table 1 and Supplementary Figure S1). The grouping was intended to emphasize the diverse mortality patterns in forests growing under contrasting climate conditions. Recent studies have shown that temperature and drought have divergent effects on high elevation *vs*. low elevation forests, as well as on temperature-limited *vs.* water-limited forests (Jolly et al., 2005; Lindner et al., 2010; Sarris et al., 2011). For Scots pine in the inner-Alps in Switzerland, mortality was found to be highest below 1000 m a.s.l., which was also related to drought (Rigling et al., 2013). We divided plots so that both ecoregions per species contained approximately equal number of plots. The resulting ecoregions encompassed low and high-altitude plots for pine (threshold at 1000 m) and spruce (1300 m). The grouping of pine and spruce by elevation resulted also in significantly different climate conditions with warmer temperatures in the lowlands, and for spruce also drier conditions, compared to the highlands (Table 1). For beech, oak and fir, which grow predominantly at lower elevations, we distinguished dry and wet plots based on the SWB_min_ (beech, fir SWB_min_ = 100 mm, oak SWB_min_ = 50 mm).

**Table 1:** Stand characteristics for 1960–2012 period summarizing mean values and the 0.05–0.95% quantile range (in parentheses) for plots within a given species-ecoregion.

Species	Ecoregion	Altitude (m a.s.l.)	SWB_min_ (mm)	Temp (°C)	Precip (mm)	BA (m^2^ ha^-1^)	Height (m)	mDBH (cm)	Mortality (% yr^-1^)
Pine	Low	680a^∗∗∗^ (509–763)	80 37–134)	15.3a^∗∗∗^ (10.2–16.2)	471 (378–582)	34.5 (14.2–58.1)	14.6a^∗^ (10.7–32.9)	16.9 (13.1–35.3)	2.3a^∗∗^ (0.4–7.3)
	High	1833b (1420–2074)	87 (75–128)	8.8b (8.3–10.4)	482 (406–973)	31.5 (16.8–47.5)	11.2b (11.4–26.0)	19.0 (16.7–43.0)	1.0b (0.2–3.1)
Spruce	Low	758a^∗∗∗^ (440–1155)	142a^∗∗^ (81–211)	13.3a^∗∗^ (7.9–16.1)	592 (378–821)	37.0 (19.6–58.5)	19.6 (7.1–22.7)	22.6a^∗^ (10.5–23.4)	1.3a^∗∗^ (0.5–3.3)
	High	1536b (1341–1835)	104b (66–172)	11.1b (8.3–15.7)	564 (404–973)	39.9 (27.6–52.9)	18.5 (6.8–22.0)	27.4b (11.5–36.8)	0.8b (0.2–2.1)
Fir	Dry	674 a^∗∗^ (520–867)	59a^∗∗∗^ (22–95)	16.0a^∗∗∗^ (14.4–16.4)	425a^∗∗∗^ (415–468)	34.5a^∗^ (19.1–41.7)	13.0a^∗^ (6.8–20.1)	22.2 (15.5–27.8)	2.1 (1.1–3.6)
	Wet	899b (404–1203)	162b (122–211)	12.5b (7.9–15.9)	631b (455–738)	42.4b (32.8–61.2)	18.4b (9.5–31.4)	26.2 (13.2–41.0)	2.0 (0.3–4.1)
Beech	Dry	587 (450–736)	83a^∗∗∗^ (59–109)	15.3a^∗∗∗^ (9.9–16.4)	447a^∗∗∗^ (378–512)	31.5a^∗^ (19.7–45.4)	18.5a^∗^ (11.8–25.5)	20.0a^∗^ (14.0–28.0)	1.4 (0.0–5.0)
	Wet	611 (430–1143)	151b (151–218)	13.5b (7.9–16.1)	590b (445–907)	35.3b (22.0–49.5)	21.4b (11.6–34.2)	23.1b (11.2–38.0)	1.2 (0.2–3.1)
Oak	Dry	607a^∗^ (436–877)	50a^∗∗∗^ (14–81)	16.0a^∗∗∗^ (15.6–16.4)	422a^∗∗∗^ (373–461)	26.8 (17.8–37.9)	16.9a^∗^ (7.8–26.7)	17.6 (8.9–27.7)	2.1 (0.1–5.3)
	Wet	543b (420–756)	127b (81–188)	13.5b (6.7–16.2)	522b (432–912)	27.2 (15.8–40.3)	19.9b (8.4–28.4)	19.1 (11.1–28.0)	2.1 (0.2–3.9)

*SWB_*min*_, minimum Site Water Balance during the growth season; Temp, mean temperature during the growth season; Precip, mean total precipitation during the growth season; BA, basal area; mDBH, stand mean DBH as indicator of stand age; Mortality, annual mortality rate. Letters (‘a’, ‘b’) indicate differences in climate and stand characteristics between ecoregions per species, tested by an unpaired t-test. Significant coefficients are indicated as follows ^∗∗∗^*P <* 0.001, ^∗∗^*P <* 0.01, ^∗^*P <* 0.05.*

### Mortality Rates

Based on the individual tree data, annual mortality rates *m* were calculated at a population level per inventory period (interval) as follows:

(1) $$m=\left(1-\left({\left(\frac{N_{\text{t}}}{N_{\text{0}}}\right)}^{\frac{1}{t}}\right)\right)\times 100$$

where *N_0_* and *N_t_* are the numbers of living trees at the beginning and end of the interval, respectively, and t is the inventory interval in years (Sheil and May, 1996). Mortality rates *m* were only calculated for populations with N_0_ > 10. In populations with different mortality probabilities, mortality rates decline with the length of the inventory interval, because the fraction of trees with a higher mortality probability declines faster than the fraction of trees with a low mortality probability. Therefore, mortality rates calculated from diverse interval lengths were compared for subpopulations for which homogeneous mortality probabilities may be assumed (Sheil and May, 1996). To account for the different interval length in our data set, we followed the approach adopted by Rohner et al. (2012) and calculated mortality rates for subpopulations, i.e., for each tree species and for three DBH-classes (small, medium, large) separately, assuming homogeneous mortality probability for these subpopulations. DBH-classes for each plot and each time period were defined so that all three DBH-classes contained the same number of trees.

To allow for comparison among plots, we standardized the mortality rate *m* per plot and inventory period by dividing it by the mean mortality rate of the plot during the total observation period:

(2) $$rm_{\text{i}}=\frac{m_{\text{i}}}{\text{mean}(m)}$$

where *rm* is the normalized mortality (%), *m* is the mortality rate as in Equation (1) and _i_ is the inventory period.

### Statistical Analyses

**FIGURE 2:**
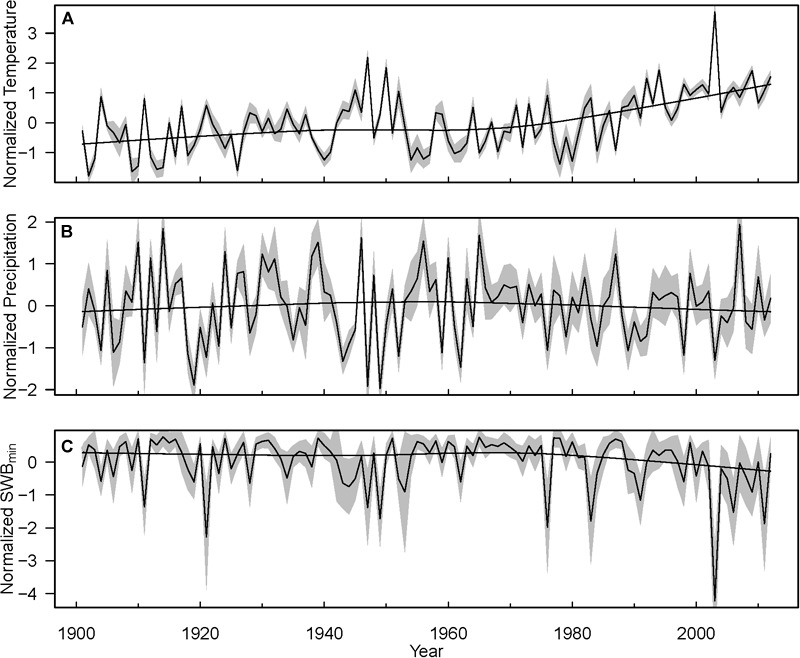
Mean trends of normalized annual temperature **(A)**, precipitation **(B)**, and SWB_min_ **(C)** during growth period for the study sites during the years 1901–2012. Yearly data were normalized by their long-term average. Black lines with gray areas indicate the mean ± SD. For a better trend visualization loess smoother was added.

We distinguished two time periods: the full time period covered by the inventory data set of 1898 to 2013, and a shorter time period including only the recent decades since 1960. This was done because (i) many plots (especially within the LWF and SNR networks) were established later than 1960, and (ii) climate in Switzerland started to change around 1960 (Figure 2). Hereby, inventory data were available for the period 1898–2013 and climate data for the period 1901–2012. Therefore, for analyses based only on inventory data, we considered the whole length of the inventory time series, whereas for analyses including also climate data we used inventory data only for 1901–2012 to match with the time-period of climate data availability.

To assess the temporal trend of mortality, generalized linear mixed effects models (GLMM) for proportions with annual mortality rate as response variable were used, i.e., logistic regression models assuming binomial distribution (Zuur et al., 2009). Random effects were defined for the intercept with study plots nested in forest sites (see “Study sites” section) as grouping factor. We used two different model approaches: (1) We fitted one model per species and ecoregion with year at the beginning of the inventory period and DBH-class as fixed effects (Equation 3). (2) In order to detect whether mortality within the three DBH-classes followed different (e.g., counteracting) trends, we fitted additional GLMMs for the three DBH-classes separately (Equation 4).

(3) $${\text{Y}}_{\text{ijk}}\text{=}\alpha \text{+}\beta_{\text{1}}\times {\text{Year}}_{\text{ijk}}\text{+}\beta_{\text{2}}\times {\text{DBH-class}}_{\text{ijk}}{\text{+a}}_{\text{j}}{\text{+a}}_{\text{j|k}}\text{+}\varepsilon_{\text{ijk}}$$

Where Y*_ijk_* is the annual mortality rate per observation i, study site j and study plot k, α is the intercept, Year is the start year of inventory, and DBH-class is a factor with three levels (small, medium, large). Fixed effects parameters are given as β, with β_1_ indicating the temporal trend of the mortality time series and β_2_ indicating the additive effect of DBH-class. a*_j_* and a*_jjk_* are site and plot random effects, assuming normal distribution with mean = 0 and σ_j_^2^ and σ*_jjk_* ^2^, respectively.

(4) $$Y_{\text{ijk},}{}_{\text{DBH-class}}\;=\alpha +\beta_{\text{1}}\times Year_{\text{ijk}}+a_{\text{j}}+a_{\text{j|k}}+\varepsilon_{\text{ijk}}$$

where Y*_ijk,DBH-class_* is the annual mortality rate per observation i, study site j and study plot k of a given DBH-class (small, medium or large). All other parameters are the same as described for Equation 3.

Multivariate relationships between explanatory variables and temporal changes in mortality rates were analyzed using GLMMs as described for Equations 3 and 4, but considering a pool of 8 initial predictors (stand characteristics, plot properties, and climate variables) and 4 interactions as fixed effects (Equation 5). All possible combinations of models were calculated per species and ecoregion. To avoid oversaturation of a model, a maximum of 5 (fir) and 7 (other species) explanatory variables were included simultaneously (resulting in 310 and 560 potential models, respectively). Preliminary analysis indicated that an additional error structure to account for plot spatial autocorrelation did not improve model performance and was not incorporated into the final model.

(5) $$Y_{\text{ijk}}=\alpha +\beta_{\text{1}}\times x_{\text{1ijk}}+\beta_{\text{2}}\times x_{\text{2ijk}}+\cdots +\beta_{\text{n}}\times x_{\text{nijk}}+a_{\text{j}}+a_{\text{j|k}}+\varepsilon_{\text{ijk}}$$

where x*_1…_*x*_n_* are the included explanatory variables as described below. All other parameters are the same as described for Equation 3.

We considered the following initial predictors: Basal area (BA, m^2^ ha^-1^) was recorded at the beginning of each inventory period and indicated the competitive state in the stand. As stand age was not available for each plot, we used the mean stand DBH as a proxy for stand age (mDBH, cm). Plot-wise regressions of mDBH against stand age for 100 plots with available stand age information resulted in a median adj. *R*^2^ of 0.96. Further, we included the DBH-classes and topographic properties, such as altitude (m), slope (degree) and aspect (as factor with four levels) as fixed effects. Mean temperature (Temp, °C) and SWB_min_ (mm) as drought indicator during the growth period were calculated for all inventory periods and then related to the long-term average (ΔTemp, ΔSWB_min_). All numerical variables (BA, mDBH, slope, altitude, ΔTemp, ΔSWB_min_) were standardized. We tested for interactions between BA and/or mDBH and the climate variables (ΔTemp and ΔSWB_min_). Only plots with at least two inventory periods available were included in the GLMMs (*n* = 224). To account for collinearity of explanatory variables only models with a maximum correlation coefficient between explanatory variables smaller than 0.7 were considered. Models were ranked according to their corrected AIC, AIC*c* (Hurvich and Tsay, 1989). In case of several comparable models (Akaike weight of the best model < 0.9) an average model of all models with a ΔAIC*c* (calculated from the model with lowest AIC*c*) < 4 was calculated (Burnham and Anderson, 2002). Model residuals were visually checked for heterogeneity *vs*. fitted values and included variables.

All statistical analyses were performed in R statistical software (R Development Core Team, 2011) with the function glmer (package ‘lme4’) for GLMMs and dredge and model.avg (package ‘MuMin’) for model selection and model averaging, respectively.

## Results

### Overview of Annual Mortality Rates

**FIGURE 3:**
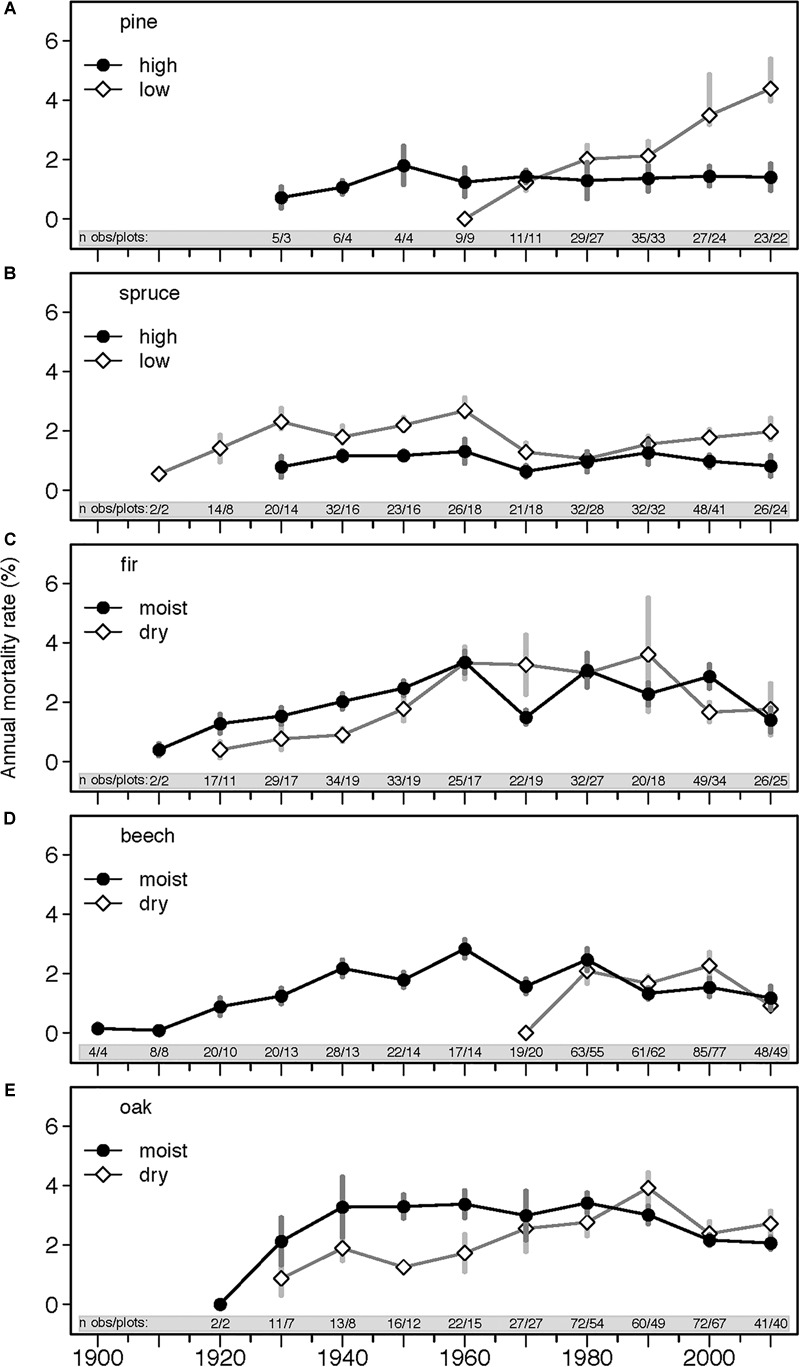
Annual mortality rates (%) for the five studied species (**A**, pine; **B**, spruce; **C**, fir; **D**, beech; **E**, oak) and ecoregions across the entire study period (1898–2013). Each data point represents mean annual mortality rate ± SE calculated at 10-year intervals and averaged across all measured plots. Numbers in the gray band (‘n obs/plots’) indicates the number of observations included/number of plots monitored during the respective inventory interval. Inventory intervals ranged from 3 to 22 years and were carried out in different years for different plots. Because of the different inventory intervals and dates, the temporal trajectories can differ from results shown in Table 2.

Average annual mortality rate for all species across the ∼120 years was 1.5%, and a considerable variation in mortality rates across species and ecoregions (dry and wet, high and low altitudes) was observed (Table 1 and Figure 3). Oak and fir had highest mean annual mortality rates of 2 and 1.8%, respectively, pine and beech had 1.3%, and spruce had the lowest mortality rate of 1% (Table 2). However, mortality rates of individual plots ranged from 0% to over 13%, with majority of plots (95%) averaging below 6%.

**Table 2:** Mean annual mortality rates per species ± standard error (SE) calculated for different study periods.

	1898–2013		1898–1960		1960–2013	
	Mean ± SE	*N*^∗^	Mean ± SE	*N*^∗^	Mean ± SE	*N*^∗^
Pine	1.3 ± 0.18	70	1.3 ± 0.33	4	1.4 ± 0.19	70
Spruce	1.0 ± 0.10	58	1.7 ± 0.18	16	1.1 ± 0.11	58
Fir	1.8 ± 0.16	37	1.8 ± 0.20	19	2.0 ± 0.21	37
Beech	1.3 ± 0.13	100	1.5 ± 0.19	14	1.3 ± 0.13	100
Oak	2.0 ± 0.18	78	1.7 ± 0.36	17	2.1 ± 0.18	78
Mean	1.5 ± 0.08	276	1.7 ± 0.14	51	1.6 ± 0.09	276

*N, number of plots. ^∗^Sum of N per species is higher than overall plot number since more than one species occurred in some plots.*

### Driving Factors of Annual Mortality Rates

Out of an initial set of 8 predictor variables and 4 interactions, GLMMs were calculated for each species and ecoregion to identify the drivers of temporal variation in tree mortality (Table 3), which are presented in detail in the following.

**Table 3:** Coefficients of the averaged generalized mixed effects models (GLMM) with mortality rate as response variable and stand characteristics, climate and topography as explanatory variables, as well as interactions of stand characteristics with climate variables (indicated by ‘:’).

													mDBH:	BA:	mDBH:	BA:
Species	Ecoregion	DBH-class		BA	mDBH	ΔSWB_min_	ΔTemp	Altitude	Slope	Aspect			ΔSWB_min_	ΔSWB_min_	Temp	Temp	N models
		Medium	Large							*E*	*S*	*W*					
Pine	Low	-0.441*	-0.393*	*0*.*302*	0.366	-0.562***		0.677***					0.638**				5
	High	-0.504***	-0.839***	0.291**	*0*.*182*		-0.181*				*0*.*422*	0.943***	-0.191**		-0.327***		6
Spruce	Low	-0.766***	-1.300***	0.608***	-0.203***	-0.154***	-*0*.*023*						-0.279***		-0.269***		1
	High	-0.518***	-0.311**	*0*.*173*	0.346**	-*0*.*005*	-*0*.*038*							-0.091*		-0.171***	14
Fir	Dry	-0.546***	-0.168*	1.401***	0.389***		-0.190***		-1.129***						-0.138*		2
	Wet	-0.262***	-0.121*	0.328***				-0.246*									31
Beech	Dry	-0.999***	-1.233***	0.199*		0.161 .				*0*.*328*	-*0*.*118*	-0.530*					25
	Wet	-1.431***	-2.104***	0.837***	-0.711***	*0*.*056*	-*0*.*038*	-0.452**	-0.306**	-1.548***	-1.350**	-0.696*	-0.112*	0.082**	0.115**	-0.117***	6
Oak	Dry	-1.122***	-2.102***	2.060***	0.698***	0.724***	-0.818***						-0.473***				2
	Wet	-1.297***	-2.242***	0.331***	-0.382***	0.452***	0.339***								-0.529***	-0.291***	1

*DBH, diameter at breast height; mDBH, stand mean DBH as indicator of stand age; BA, basal area; ΔSWB_*min*_, deviation from the long-term mean SWB_*min*_ (minimum Site Water Balance) during the growth season; ΔTemp, deviation from the long-term mean temperature during the growth season. DBH “small” and exposition “N” (North) were set as reference level. E, East; S, South; W, West. Significant coefficients are indicated as follows ^∗∗∗^*P <* 0.001, ^∗∗^*P <* 0.01, ^∗^*P <* 0.05, ‘.’*P <* 0.1. Coefficients of variables that were non-significant as single predictors, but were included as components in a significant interaction are displayed in italics.*

### Stand Properties

**FIGURE 4:**
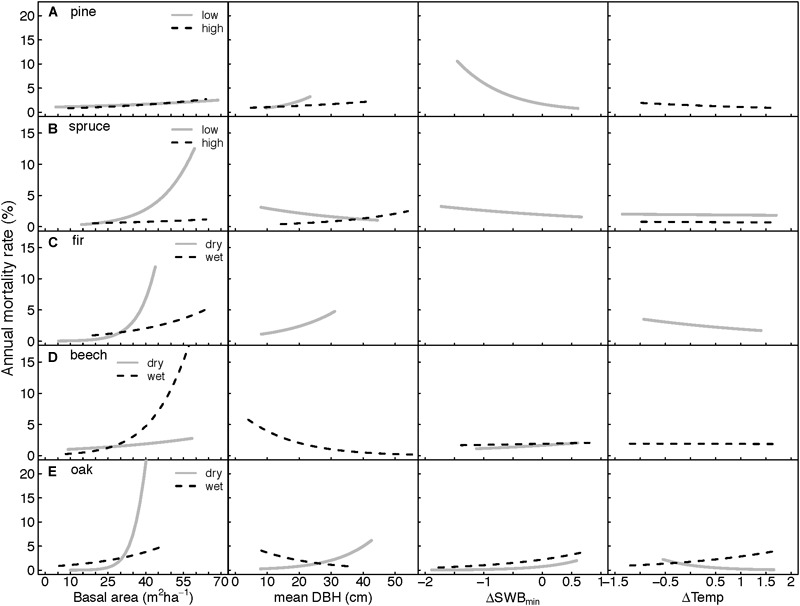
Curves illustrating predicted response of annual mortality rates to stand and climate variables as derived from the GLMMs (Table 3). **(A–E)** Correspond to the five species studied. **(A)** pine, **(B)** spruce, **(C)** fir, **(D)** beech, and **(E)** oak. Response curves were calculated by holding all predictors in the model constant at their mean, except for the selected displayed variable. Curves represent a mean response over the three DBH-classes, and four aspect levels (if significant in the model). ΔSWB_min_: deviation from the long-term mean SWB_min_ (minimum Site Water Balance) during the growth season, ΔTemp: deviation from the long-term mean temperature during the growth season.

Basal area (BA) was the most important and consistent predictor of annual mortality rates for all species and ecoregions. Increasing annual mortality rates were correlated with higher BA (Table 3 and Figure 4). The strongest effect was observed for fir and oak growing on dry sites and beech from wet sites. BA was not significant for only two species-ecoregions, pine at low altitudes and spruce at high altitudes. In the latter case, however, interaction of BA with climate variables was significant (Table 3).

Mean DBH (mDBH, proxy for stand age) had a significant effect in six out of the ten species-ecoregion combinations and both negative and positive relationships were observed (Table 3). Increased mortality rates were associated with higher mDBH in pine from low altitudes, spruce from high altitudes, and fir and oak both from dry regions, while for spruce at low altitudes, and oak and beech in wet regions, increased mortality rates were related to lower mDBH. Annual mortality rates varied considerably between the three DBH-classes. Overall, smallest trees had the largest annual mortality rates, while largest trees had, in most cases, lower mortality rates (Table 3).

### Climate Conditions

Changes in the minimum site water balance (ΔSWB_min_) and in temperature (ΔTemp) had variable effects on annual mortality rates, and were significant in four species-ecoregions for ΔSWB_min_ and for ΔTemp (Table 3 and Figure 4). Increasing temperatures were associated with increasing mortality only in oak from wet regions, while mortality decreased in pine at high altitudes, fir and oak both in dry regions. The strongest negative impact of ΔSWB_min_ on annual mortality rates was recorded for pine in the lowlands and a weaker effect for spruce in lowlands, thus mortality increased under drier conditions. In contrast, for pine and spruce at high altitudes no significant impact of ΔSWB_min_ on annual mortality rates was found. Fir mortality was not related to ΔSWB_min_ in neither of the two ecoregions. For beech and oak, mortality rates were positively associated with ΔSWB_min_, indicating an increase in mortality under wet conditions. For beech at dry sites the relationship was only marginally significant, however, ΔSWB_min_ was included in 24 of the best 25 models. Although the relationship of ΔSWB_min_ and mortality was not significant in the averaged model for beech at wet sites, ΔSWB_min_ was included in the best ranked models either as significant single parameter or in a significant interaction (Supplementary Table S2). For both, beech and oak, mortality rates at a given ΔSWB_min_ appeared to be slightly higher on wet sites compared to dry sites (Figure 4), for beech especially in denser stands (cf. Supplementary Figure S3), but differences were generally small. Larger differences were observed for ΔTemp. For beech, only wet sites showed a negative temperature effect on mortality in interaction with stand age, whereas mortality was not related to ΔTemp on dry sites. For oak, mortality was increased at higher ΔTemp on wet sites, whereas the sign was reversed at dry sites (Figure 4).

The impact of ΔSWB_min_ and/or ΔTemp on mortality was dependent on mDBH for all species, particularly at low and dry sites (Supplementary Figures S2, S3). In general, the effect of ΔSWB_min_ was stronger in old stands, except for pine at low altitudes with a reversed interaction. The effect of ΔTemp negatively interacted with mDBH, indicating a greater vulnerability of young stands relative to old stands to increased temperature. At high-altitude and wet sites, the magnitude of the climatic effect (ΔSWB_min_ and/or ΔTemp) on mortality was stronger for dense forests stands (i.e., higher stand BA) (Table 3 and Supplementary Figure S3). This was consistent for all species.

### Altitude and Site Topography

Altitude, slope and aspect were only occasionally related to mortality (Table 3) with no clear pattern. For instance, for beech growing on wet sites, mortality rates were negatively correlated with altitude, while for pine from low altitude regions, the pattern was opposite. The steepness of the slope was significantly related to mortality for beech from wet and fir from dry regions. Aspect was negatively related to mortality rates for beech from both dry and wet regions and positively in pine from high altitudes. For all other species-ecoregions the relationships of mortality with topography were not significant.

### Temporal Trends of Annual Mortality Rates

Temporal trends of annual mortality rates varied considerably across species and ecoregions throughout the entire study period (starting in ∼1900s, 1910s, 1920s, and 1930s depending on the species and ecoregion). Generalized mixed effect models indicated that over the last ∼120 years, pine and spruce from high altitudes and fir from dry regions exhibited the highest increase of annual mortality rates by 1.6, 1.0, and 1.3% per year, respectively (Table 4). For other species and ecoregions either no significant or very small shifts were observed (<±0.5%). For pine in lowlands and beech in dry regions, time series started later than 1960 and were therefore not included in this analysis.

**Table 4:** Results of the GLMMs with annual mortality rate as response variable, with first year of the inventory period as fixed effect, and plot nested in forest site (see Materials and Methods, Equations 3 and 4) as random effect.

			DBH-class			
		Time
Species	Ecoregion	period	Small	Medium	Large	Average
Pine	Low	1898–2013	NA	NA	NA	NA
		1960–2013	0.023.	0.033*	n.s.	0.023**
	High	1898–2013	n.s.	0.015**	0.015*	0.016***
		1960–2013	n.s.	n.s.	n.s.	n.s.
Spruce	Low	1898–2013	-0.003**	n.s.	0.007**	n.s
		1960–2013	0.013**	n.s.	0.025.	0.018***
	High	1898–2013	0.007*	n.s.	0.012***	0.010***
		1960–2013	n.s.	n.s.	0.036***	0.016***
Fir	Dry	1898–2013	0.010***	0.014***	0.015***	0.013***
		1960–2013	-0.034***	-0.021***	n.s.	-0.018***
	Wet	1898–2013	n.s.	0.004***	0.007***	0.004***
		1960–2013	-0.012***	-0.006***	0.015**	n.s.
Beech	Dry	1898–2013	NA	NA	NA	NA
		1960–2013	n.s.	0.005***	0.023.	n.s.
	Wet	1898–2013	-0.007***	n.s.	0.007*	-0.004***
		1960–2013	-0.023***	n.s.	0.030***	-0.009***
Oak	Dry	1898–2013	n.s.	0.007***	0.015*	0.005*
		1960–2013	-0.054***	-0.032***	n.s.	-0.042***
	Wet	1898–2013	n.s.	n.s.	n.s.	n.s.
		1960–2013	-0.005***	-0.028***	-0.070***	-0.011*

*Models were calculated for each DBH-class (small, medium, large) separately, as well as for all trees together. For the latter the DBH-class was additionally included in the model as fixed effect. ‘Low’ and ‘High’ refers to low and high altitudes. NA, not enough data available for period before 1960. Significant coefficients are indicated as follows ^∗∗∗^*P <* 0.001, ^∗∗^*P <* 0.01, ^∗^*P <* 0.05, ‘.’*P* < 0.1, n.s., not significant.*

Throughout 1960–2013, pine in lowlands showed the highest increase of mortality rates by 2.3% per year, followed by spruce in both ecoregions, with an increase by 1.8 and 1.6% per year at low and high altitudes, respectively (Table 4). Fir from dry regions, beech from wet regions and oak from both dry and wet regions showed decline in mortality rates with most pronounced shift in oak from dry regions (-4.2% per year). In the remaining three species-ecoregions the change was not significant.

**FIGURE 5:**
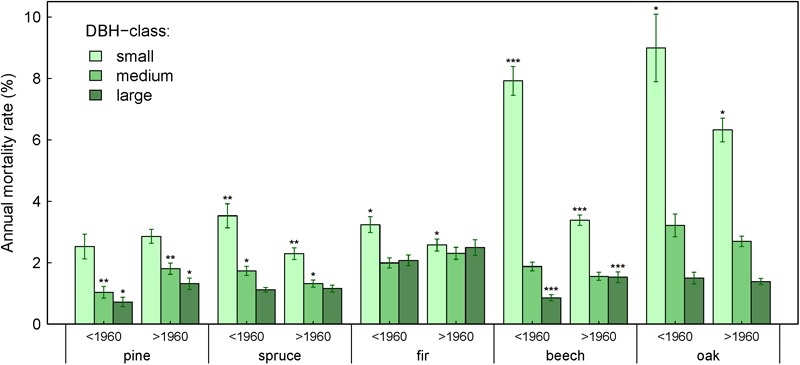
Annual mortality rate by species and DBH-class for time periods before and after 1960. Asterisks indicate statistically significant differences in mortality rates per DBH-class and species between the two time periods (^∗∗∗^*P* < 0.001, ^∗∗^*P* < 0.01, and ^∗^*P* < <0.05, unpaired *t*-test on normally distributed data). Number of plots included is the same as in Table 2.

Over the last ∼120 years, mortality of trees within the large and small DBH-classes exhibited opposite trends: mortality of large trees increased in seven species-ecoregions, while mortality of small trees decreased, increased or showed no significant trend (Table 4). Since 1960, this tendency became even more pronounced with an increase of mortality in large trees for six species-ecoregions, and a decrease in small trees for five species-ecoregions or no significant changes. Exceptions were fir at dry sites, where all DBH-classes showed increases in mortality, and in oak where a general decrease of mortality across the DBH-classes was observed. These opposing tendencies also emerged when comparing mortality rates of DBH-classes between two periods before and after 1960, although changes were not always significant due to large data heterogeneity (Figure 5).

**FIGURE 6:**
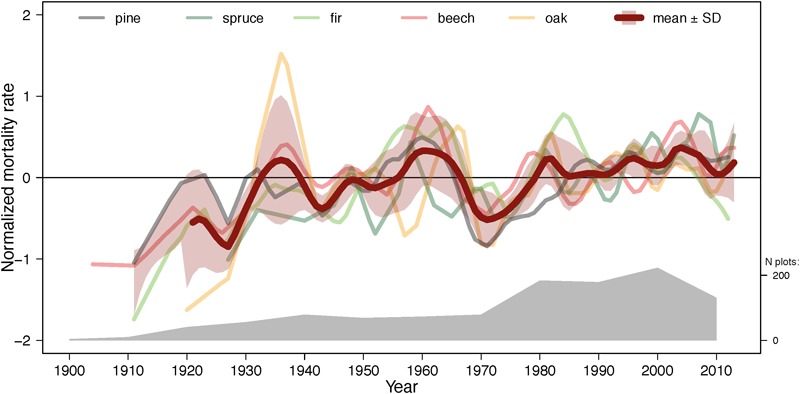
Normalized mortality rates averaged across all species (bold line) and individual species (thin lines) over the entire study period. The lines are smoothed with a spline function. Normalized mortality rate is annual mortality rate divided by average mortality rate across the entire study period. The misalignment of trajectories may be partly due to different inventory dates and intervals. The number of plots is indicated by the gray polygon (see also Figure 3).

Despite notable differences between species in temporal trends of annual mortality (Figure 3 and Table 4) and heterogeneous data (different number of plots, inventory dates and intervals between the inventories), the five species displayed similar fluctuations in normalized mortality rates (Figure 6 and Supplementary Table S3). Normalized mortality rates express annual mortality rate relative to the long-term average annual mortality rate. Although annual mortality rates changed only mildly during the last ∼120 years (Table 4), it is noteworthy that in the most recent three decades (1980–2010) the cross-species average mortality rate did not drop below the 100-year average. In addition, the frequency of mortality peaks in the last three decades was also higher than earlier. The magnitude of the peaks, however, decreased probably due to the higher plot number in recent years. There were also distinct differences in the temporal mortality trends (cf. Table 4). For instance, the temporal trajectory of beech mortality correlated well with all four other species (Figure 6 and Supplementary Table S3), while trajectory of oak mortality was well associated with only two other species: beech and pine.

## Discussion

In this study, we assessed mortality rates of five dominant species in Swiss forests throughout the last century (1898–2013) and examined factors driving mortality change. The complex study design with different points in time of forest inventories as well as differing length of the inventory periods within and across the three monitoring networks challenges statistical analysis and generates limitations on data interpretation. Nevertheless, some very clear patterns emerged from the one century-long data set. Since 1960, mortality rates of pine in lowlands and of spruce increased significantly, whereas those of oak trees decreased (Table 4). Fir and beech showed only minor or not significant changes in mortality rates. Stand properties, especially BA, were the most important factors influencing variability of mortality rates, while climate impact (temporal changes in minimum site water balance and temperature) was inconsistent across species and ecoregions, and depended on interactions with mDBH (i.e., an indicator of stand age) and BA (indicating competition) (Table 3). Thus, we could not conclusively confirm our initial hypothesis, that oak and pine would be most tolerant, and beech and spruce would be most vulnerable to drought, at least not in absence of other stand-related factors. The impact of drought and heat on mortality appeared rather to result from a combination of species and site effects, and being further modulated by competition and stand age. The long-term data presented here highlight the complex nature of the processes shaping forest dynamics. Throughout one century, unmanaged forests gradually alter their structure, demography and in some cases also their species composition. Consequently, structural and demographical changes in combination with climate affected mortality rates in our study, implying that a variety of factors need to be accounted for in mortality studies at the ecosystem scale. Below, we discuss the annual mortality rates, the effect of competition and climate variability on mortality, and its impact on the future development of forests in Switzerland.

### Annual Mortality Rates

Average annual mortality rate through the entire study period was 1.5% but varied considerably across species and plots (Figure 3 and Tables 1, 2). On average, mortality rates of various tree species in Europe range between 0.1 and 2.9 %, with a median of 0.5% (Supplementary Table S5), which is lower compared to mortality rates in this study. Beyond the overall inherent, high variability of mortality rates across forests, this may be due to several methodological and conceptual reasons: (1) the 5 cm DBH threshold applied here was lower than in other studies, and this might have increased the average mortality rate of the investigated populations, since mortality tends to be higher in small trees (Rohner et al., 2012; Rigling et al., 2013); (2) The data set included a high proportion of young stands, for which mortality rates are usually higher compared to forests in the optimum phase (Oliver and Larson, 1996); (3) The long study period of over 100 years may have increased the probability of mortality events, although fluctuations of mortality rates across different decadal time periods were small (Supplementary Table S4); (4) Our study encompassed a large number of plots across broad altitudinal and climate gradients, including also plots at species distribution margins with exceptionally high mortality, rising the overall average mortality rates; (5) Finally, the absence of management in our study sites might explain the relatively high mortality rates, especially in small and medium size tree classes. Thus, a direct comparison of mortality rates across different studies should be interpreted with caution. These and other factors challenge a direct comparison of absolute mortality rates across different studies, while relative changes of mortality rates as well as their driving factors should be comparable across studies.

### Drivers of Mortality

Mortality rates were best explained by a combination of stand properties and climate variables, whereas altitude and topography were less significant. In concordance with other studies (Condés and del Río, 2015; Pillet et al., 2018) our results showed that when climate and competition considered together a larger portion of variation in mortality rates can be explained, as when considered separately.

### Effects of Stand Characteristics on Mortality Rates

Basal area, and thus competition, was the strongest predictor variable of mortality rates. For all species and ecoregions a consistent effect of increasing mortality with higher BA was found (Table 3 and Figure 4), as high stand density leads to increased competition for resources, which consequently may result in higher mortality (Giuggiola et al., 2013; Ruiz-Benito et al., 2013; Condés and del Río, 2015; Bradford and Bell, 2017; Young et al., 2017).

In all species, mortality rates were largest among the smallest diameter trees, while the largest trees had lowest mortality rates, as reported also in many other studies (Monserud and Sterba, 1999; Mantgem and Stephenson, 2007; van Mantgem et al., 2009; Peng et al., 2011; Neumann et al., 2017) and could be explained by smaller trees being outcompeted by larger trees (Coomes and Allen, 2007) or by trees of the same size class. The impact of stand age (implied by mean DBH) on mortality rates was significant for most species and ecoregions, but with variable sign and strength, which might reflect differences in stand development of the included plots.

### Effects of Climate on Mortality Rates

Climate variables, i.e., changes in temperature and SWB_min_, had contrasting influence on mortality rates, depending on species and ecoregion. For spruce and pine, mortality of lowland forests increased with increasing dryness (negative ΔSWB_min_). Contrary to our initial hypothesis, this effect was most conspicuous for pine, for which SWB_min_ was the overall strongest predictor of annual mortality rates (Table 3 and Figure 4). Increased mortality of pine due to drought became, however, an increasingly observed pattern in the dry valleys of the Valais, in Grisons and the Churer Rheintal in Switzerland (Dobbertin et al., 2005; Bigler et al., 2006; Rigling et al., 2006, 2013; Wohlgemuth and Rigling, 2014). Although pine can recover after a few incidents of drought (Eilmann et al., 2010; Eilmann and Rigling, 2012), several consecutive dry years can lead to a reduction in vitality and associated higher susceptibility of trees to pests, pathogens and parasites (Bigler et al., 2006; Rigling et al., 2010). Although Scots pine is generally drought-tolerant, but - growing already on dry sites – an increase in frequency and severity of drought events may force it beyond its physiological limits. Given that pine has been introduced by humans outside its native range and was promoted until 1950 (Gimmi et al., 2010), it is now being outcompeted by indigenous or invasive species, or species which are better adapted to dry conditions, like pubescent oak (Rigling et al., 2013). Spruce in the lowlands has also been found to be susceptible to drought (Vanoni et al., 2016a,b) and is expected to be substituted by more drought-tolerant species in the future (Hanewinkel et al., 2013). However, we did not find a very strong impact of drought on spruce mortality. Instead, BA and mDBH were more influential. This might be due to the fact that we excluded plots that were substantially affected by storm damages or bark beetle attacks. These plots might have been also experienced severe drought beforehand since the intensity of the attack is often amplified by preceding drought events (Meier et al., 2014; Kolb et al., 2016). Thus, secondary factors, such as bark beetles, affect spruce mortality rates often to a larger extent than drought itself. Additionally, in contrast to the lowland pine plots, which were restricted to very dry regions, lowland spruce plots exhibited on average a relatively high soil water availability (Table 1). Contrary to the lowlands, mortality rates of pine and spruce at higher altitudes were not related to drought, but increased under low temperatures, probably due to direct low-temperature limitation (Körner, 1998, 2003; Vanoni et al., 2016a) combined with indirect temperature stress such as frost damage, winter desiccation, or low-temperature photo-inhibition (Barbeito et al., 2012). Similar patterns were also observed for Scots pine growing at higher altitudes in Spain (Ruiz-Benito et al., 2013).

For silver fir trees, drought did not show any significant influence on annual mortality (Table 3). This pattern confirms other studies from the Churer Rheintal in Switzerland, where the detrimental impact of drought on growth of silver fir trees is mitigated by deep soils with higher water holding capacity and northern exposition (Wohlgemuth and Rigling, 2014). On drier sites, the strong impact of low temperatures on mortality rates, could presumably be explained by the sensitivity of silver fir to frost (Lebourgeois et al., 2010). In summary, silver fir in Switzerland seems to be able to cope with the current level of experienced droughts and is still within its physiological boundaries, as also observed in other parts of Europe (George et al., 2015).

Increasing mortality with higher water availability was found for beech and, even more noticeably, for oak (Table 3 and Figure 4). This pattern is counter-intuitive, especially for beech on dry sites, although it was also observed in other studies (Rohner et al., 2012; Nothdurft, 2013; Ruiz-Benito et al., 2013), and might be related to other concurrent abiotic conditions. Presumably, higher water availability in moist years can lead to an increase of total leaf area and subsequent overshadowing of short, young trees limiting their access to light and water, consequently resulting in their increased mortality. Beech is often considered to be drought sensitive (Gessler et al., 2007) and, in general, performs better on wetter sites (Aranda et al., 2000; Gessler et al., 2007). A recent study also found that beech growth appears to be very location specific, with growth decline at lower altitudes and growth increase at higher altitudes (Dulamsuren et al., 2017). In concordance with this, we observed higher beech mortality at lower altitudes compared to higher altitudes.

It has been observed that beech (Vitasse et al., 2014) and also oak (Jensen and Hansen, 2010; Grossiord et al., 2014) trees can adapt very well to dry conditions, possibly due to their deep rooting penetration, efficient stomatal control (Nothdurft, 2013), and high degree of evolutionary adaptability (Roloff and Grundmann, 2008). Thus, higher mortality rates per given ΔSWB_min_ at wet sites compared to dry sites for beech and oak partly confirm the hypothesis that drought resistance of trees increases with lower site water availability (Kunz et al., 2018). This might be due to the high degree of genetic adaptability. However, differences related to SWB were small and might also be associated with generally higher turn-over at sites with higher water availability. The temperature effect on mortality of the two species clearly differed between dry and wet sites of the two species, indicating that trees that are adapted to dry and warm conditions might be able to cope better with occurring heat waves as projected for the future. For oak species, the impact of pathogens related to wet conditions and frost (Führer, 1998; Gaertig et al., 2005) but also to drought (Wood et al., 2018) is an additional relevant factor driving mortality (Haavik et al., 2015).

### Effects of Stand Characteristics and Climate on Mortality Rates

The inclusion of an interaction term of BA or mDBH (i.e., stand age) with climate variables improved the model parsimony for pine, spruce, fir and oak, indicating that the effect of climatic variables varies across stand structures and developmental stages (Table 3). These effects were more pronounced on dry and lowland sites compared to wet and highland sites (Supplementary Figures S2, S3). Our results indicate that the impact of drought and/or temperature on tree mortality increased with competition (i.e., higher BA), which has also been reported for many tree species in Spain (Ruiz-Benito et al., 2013). In addition, pine has been found to perform better after stand density reduction on xeric sites in Switzerland (Giuggiola et al., 2013, 2018), further indicating that competition is an important factor in driving mortality and demography (Kohler et al., 2010; Sohn et al., 2016). For age, the pattern reversed along the temperature gradient and young stands were generally more strongly affected by increased temperatures compared to old stands. This might be due to the fact that young stands are usually denser and prone to self-thinning. Our results indicate that heat and/or drought may trigger the self-thinning process in young stands.

### Temporal Trends in Annual Mortality

Temporal trends in annual mortality rates varied depending on the data grouping factor (e.g., species, ecoregions, DBH-classes, Figure 3, 5, 6 and Table 4) partly due to methodological reasons (i.e., data averaging and integration procedures). For example, discrepancies between temporal trajectories in GLMMs (Table 4) and figures (Figure 3, 5) originated from the fact that models used actual dates of inventories and interval lengths and accounted for plots as grouping factor, while figures show mortality estimates averaged per decade or even larger time periods and/or per species and ecoregions. Nevertheless, some clear patterns emerged. Species-specific trends of annual mortality rates across the ∼120 years were modest, but became larger during the second half of the century (Figure 3 and Table 4). Spruce and pine exhibited increasing mortality rates, while fir on dry sites, oak on dry and wet sites, and, to a lesser degree, beech on wet sites showed decreasing mortality rates. Thus, the normalized mortality rates (Figure 6) averaged over all species showed a slight, but consistent, increasing trend in mortality across all species, but with a large uncertainty at the beginning of the study period due to low plot number. The steady increase in the normalized mortality rates since 1900, rather than an obvious break point at the start of pronounced climatic changes around 1960 (Figure 2), suggests that the increase in mortality is more strongly driven by gradually changing stand parameters (such as density and age, as shown in the previous paragraphs) and to a lesser extent by climate. However, it is also noteworthy that fluctuations of normalized mortality rates of all species are remarkably similar, especially since the 1960s (Figure 6 and Supplementary Table S3). Thus, mortality peaks were for all species relatively (given the heterogeneity of the data structure) synchronous every 10–20 years, especially during the recent decades. Additionally, since the 1980s, annual mortality rates were consistently, although only slightly, above the long-term average. Temporal disturbance patterns strongly synchronized across landscapes have been also observed in temperate forests in Europe during 1986–2016 and were related to preceding drought and storm events (Senf and Seidl, 2018). Thus, forest stand properties, such as BA, might drive the overall long-term trend of forest mortality, while climate modulates these trends with an assumed increasing importance during the recent decades.

Our results also demonstrate that trends in annual mortality rates differed depending on tree size and even run in opposing directions. Mortality rates of small trees tended to decrease or did not change, while mortality rates of large trees generally increased over time (Figure 5 and Table 4). This might be a stand aging effect, since observed forests aged for up to 120 years throughout the study period and run through different stand developmental stages. Moreover, recent studies in temperate forests indicate that susceptibility to drought increases with tree age or size (Carrer and Urbinati, 2004; Lloret et al., 2011; Ding et al., 2017), probably due to higher risk of hydraulic failure (Bennett et al., 2015; McDowell and Allen, 2015) or higher costs involved in growing new functional xylem after the drought has passed (Trugman et al., 2018). As a consequence, an increased mortality of large trees may have allowed for higher survival of young trees. However, also contradictory observations have been made, showing higher susceptibility to drought of young trees (Mantgem and Stephenson, 2007; Colangelo et al., 2017). More research is needed to clarify this context dependency (Ding et al., 2017).

### Future Implications

Contrarily to our initial hypothesis, we found a significant drought-induced mortality only in pine at low altitudes and, to a lesser extent, in spruce from low altitudes (Table 3). It appears, that drought-tolerant pine, growing already on dry sites, was severely impacted by drought events, but that fir, beech and oak, growing mainly on soils with a good water holding capacity, seemed not yet to be affected by the occurrence of drought in Switzerland. They appear to be quite well adapted to the current conditions within their ecological niche. This pattern is contrary to many studies reporting rising mortality rates due to drought and heat (Breshears et al., 2005; Gitlin et al., 2006; Allen et al., 2010, 2015; Anderegg et al., 2013; Gustafson and Sturtevant, 2013; Holmgren et al., 2013; Reichstein et al., 2013; Bradford and Bell, 2017; Chen et al., 2018; McDowell et al., 2018). One reason for this discrepancy may stem from the fact that many studies reported mortality after a specific drought or heatwave event and/or were restricted to relatively short time intervals of several years, including a large climatic disturbance event (for example, Martínez-Vilalta and Piñol, 2002; Dobbertin, 2005; Landmann and Dreyer, 2006; Ogibin and Demidova, 2009). The inventory intervals of five or more years also may dilute the climate signal on mortality (Dobbertin et al., 2005; Huelsmann et al., 2018). Nevertheless, our results show that mortality rates of five dominant tree species in Switzerland increased only slightly over the last ∼120 years, which could mainly be related to changes in stand structure. This suggests that Swiss forests have been resilient to recent climates change so far (with the exception of some hotspots) and that instead of an abrupt transition of forests, changes in species composition might occur more gradually and subtly. Nevertheless, the amplified effect of drought and heat under competition might indicate potentially strong changes in the demographic structure and species composition of forests in the future (Ruiz-Benito et al., 2013).

It is predicted that the future will bring longer and more intense droughts in many regions across Europe (Fischer and Schär, 2010; Dai, 2013; CH2014-Impacts, 2014). The resilience of trees to climatic changes requires further investigation, for instance, how quickly and effectively trees recover from episodes of drought and heatwaves. Some studies suggest that drought tolerant species may shift to sites that are becoming more arid, as these species are already acclimated to drier conditions. This shift may eliminate species that are already at their ecophysiological limits (Lévesque et al., 2013, 2014; Rigling et al., 2013). Moreover, species specific responses to insect attacks, pest and pathogens, may shape the structure of future forests. For example, in Germany and Austria, an increase in mortality risk is predicted especially for spruce at low altitudes, while beech and oak may be more robust to climate change (Lexer et al., 2002; Nothdurft, 2013; Ding et al., 2017), which could be supported by the observations from Switzerland reported in this study.

## Data Availability

The datasets generated for this study are available on request to the corresponding author.

## Author Contributions

AR, SE, and AZ conceived the study. SE analyzed the data with support from BR, AB, AKB, NKR, and AR. SE and KZ wrote the manuscript with contribution from all other co-authors.

## Conflict of Interest Statement

The authors declare that the research was conducted in the absence of any commercial or financial relationships that could be construed as a potential conflict of interest.
