# Supplementary material for: One Century of Forest Monitoring Data in Switzerland Reveals Species- and Site-Specific Trends of Climate-Induced Tree Mortality
Source: Front Plant Sci. 2019 Mar 22;10:307. doi: 10.3389/fpls.2019.00307 (PMC6438887; doi:10.3389/fpls.2019.00307)
Supplement: Supplementary file 1 [file Data_Sheet_1.docx]

**Supplementary Material**

**One century of forest monitoring data in Switzerland reveals species-and site-specific trends of climate-induced tree mortality**

Etzold, Sophia*, Ziemińska, Kasia, Rohner, Brigitte, Bottero, Alessandra, Bose, Arun K., Ruehr, Nadine K., Zingg, Andreas, Rigling, Andreas

*Correspondence: Sophia Etzold: sophia.etzold@wsl.ch

This supplement includes 5 Tables and 3 Figures.

**Table S1**. Overview of the stand characteristics and methods of the study plots belonging to the three monitoring networks Experimental Forest Monitoring (EFM), Long-term Forest Ecosystem Research (LWF) and Swiss Nature Reserves Network (SNR).

| **Network** | **EFM** | **LWF** | **SNR** | **All** |
| --- | --- | --- | --- | --- |
| N plots | 85 | 13 | 178 | 276 |
| N forest sites | 54 | 13 | 28 | 95 |
| N plots stand type: |  |  |  |  |
| Pure | 68 | 9 | 98 | 175 |
| Mixed | 17 | 4 | 80 | 101 |
| N plots at which species occurring: |  |  |  |  |
| Pine | 10 | 3 | 47 | 60 |
| Spruce | 14 | 4 | 24 | 42 |
| Fir | 12 | 1 | 3 | 16 |
| Beech | 15 | 3 | 51 | 69 |
| Oak | 34 | 2 | 37 | 73 |
| Average length of inventory intervals (range) | 7  (3-22) | 5  (3-6) | 12  (5-20) | 9  (3-22) |
| Start of Monitoring | 1887-1900 | 1995 | Late 1940s |  |
| DBH threshold (cm) | 2 | 5 | 4 | 5 |

**Table S2.** Coefficients of the best five generalized mixed effects models (GLMM) with mortality rate as response variable and stand characteristics, climate and topography as explanatory variables, as well as interactions of stand characteristics with climate variables (indicated by ‘:’). DBH: diameter at breast height, mDBH: stand mean DBH as indicator of stand age, BA: basal area, ∆SWB_min_: deviation from the long-term mean SWB_min_ (minimum Site Water Balance) during the growth season, ∆Temp: deviation from the long-term mean temperature during the growth season. Significant effects of factors (DBH-class and aspect) are indicated with a ‘+’. Coefficients of variables that were non-significant as a single predictor, but were included as component in a significant interaction are displayed in italics. Additionally, the degree of freedom (df), the difference of the model AIC in relation to the minimum AIC (∆AIC), as well as the Akaike weight of the model is given.

| **Model** | **DBH-class** | **BA** | **mDBH** | **∆SWB_min_** | **∆Temp** | **Altitude** | **Slope** | **Aspect** | **mDBH:**  **SWB_min_** | **BA:**  **SWB_min_** | **mDBH:**  **Temp** | **BA:**  **Temp** | **df** | **∆AIC** | **weight** |
| --- | --- | --- | --- | --- | --- | --- | --- | --- | --- | --- | --- | --- | --- | --- | --- |
| **Pine low** | |  |  |  |  |  |  |  |  |  |  |  |  |  |  |
| 1 | + | 0.293 | 0.299 | -0.637 |  | 0.664 | -0.236 |  | 0.643 |  |  |  | 11 | 0 | 0.431 |
| 2 | + |  | 0.546 | -0.372 |  | 0.701 |  |  | 0.595 |  |  |  | 9 | 2.01 | 0.157 |
| 3 | + | 0.342 | 0.365 | -0.520 | 0.050 | 0.646 |  |  | 0.691 |  |  |  | 11 | 2.02 | 0.157 |
| 4 |  | 0.281 | 0.299 | -0.637 |  | 0.676 | -0.243 |  | 0.629 |  |  |  | 9 | 2.13 | 0.148 |
| 5 | + |  | 0.458 | -0.502 | 0.034 | 0.749 | -0.296 |  | 0.605 |  |  |  | 11 | 2.80 | 0.106 |
| **Pine high** | |  |  |  |  |  |  |  |  |  |  |  |  |  |  |
| 1 | + | 0.288 | 0.161 |  | -0.175 |  |  | + |  |  | -0.317 |  | 11 | 0 | 0.279 |
| 2 | + | 0.307 | 0.189 |  | -0.171 |  | -0.107 | + |  |  | -0.318 |  | 12 | 1.45 | 0.135 |
| 3 | + |  | 0.272 | -0.012 | -0.243 |  |  | + | -0.191 |  | -0.415 |  | 12 | 1.89 | 0.108 |
| 4 | + | 0.286 | 0.165 |  | -0.166 |  |  | + |  |  | -0.295 | -0.033 | 12 | 2.01 | 0.102 |
| 5 | + | 0.286 | 0.163 | 0.021 | -0.162 |  |  | + |  |  | -0.315 |  | 12 | 2.07 | 0.099 |
| **Spruce low** | |  |  |  |  |  |  |  |  |  |  |  |  |  |  |
| 1 | + | 0.608 | -0.203 | -0.154 | -0.023 |  |  |  | -0.279 |  | -0.269 |  | 11 | 0 | 0.998 |
| 2 | + | 0.551 | -0.158 | -0.125 | 0.033 |  |  |  | -0.220 |  |  | -0.183 | 11 | 14.28 | 0.001 |
| 3 | + | 0.587 | -0.159 | -0.133 |  |  | -0.567 |  | -0.201 |  |  |  | 10 | 14.99 | 0.001 |
| 4 | + | 0.589 | -0.156 | -0.138 |  |  | -0.562 |  | -0.218 | 0.028 |  |  | 11 | 16.75 | 0 |
| 5 | + | 0.582 | -0.157 | -0.133 |  | -0.066 | -0.534 |  | -0.201 |  |  |  | 11 | 16.91 | 0 |
| **Spruce high** | |  |  |  |  |  |  |  |  |  |  |  |  |  |  |
| 1 | + | 0.159 | 0.308 | 0.003 | -0.022 |  |  |  |  | -0.091 |  | -0.186 | 11 | 0 | 0.142 |
| 2 | + | 0.183 | 0.361 |  | -0.040 |  | -0.294 |  |  |  |  | -0.167 | 10 | 0.09 | 0.136 |
| 3 | + | 0.167 | 0.343 |  | -0.038 |  |  |  |  |  |  | -0.169 | 9 | 0.11 | 0.135 |
| 4 | + | 0.181 | 0.337 | -0.033 | -0.050 |  |  |  | -0.143 |  |  | -0.174 | 11 | 1.73 | 0.060 |
| 5 | + | 0.159 | 0.354 |  | -0.045 |  |  |  |  |  | -0.050 | -0.164 | 10 | 1.83 | 0.057 |
| **Fir dry** | |  |  |  |  |  |  |  |  |  |  |  |  |  |  |
| **1** | + | 1.369 | 0.3844 |  | -0.183 |  |  |  |  |  | -0.138 |  | 9 | 0 | 0.655 |
| 2 | + | 1.521 | 0.404 |  | -0.214 |  | -1.129 |  |  |  |  |  | 9 | 2.62 | 0.177 |
| 3 | + | 1.556 | 0.402 |  | -0.218 |  |  |  |  |  |  |  | 8 | 4.52 | 0.069 |
| 4 | + | 1.515 | 0.394 |  | -0.192 |  |  |  |  |  |  | -0.080 | 9 | 4.86 | 0.058 |
| 5 | + | 1.553 | 0.403 |  | -0.219 | -0.831 |  |  |  |  |  |  | 9 | 5.53 | 0.041 |
| **Fir wet** | |  |  |  |  |  |  |  |  |  |  |  |  |  |  |
| 1 | + | 0.315 |  |  |  | -0.253 |  |  |  |  |  |  | 7 | 0 | 0.076 |
| 2 | + | 0.342 | -0.065 |  |  | -0.253 |  |  |  |  |  |  | 8 | 0.74 | 0.053 |
| 3 | + | 0.311 |  |  | 0.020 | -0.252 |  |  |  |  |  |  | 8 | 1.49 | 0.036 |
| 4 | + | 0.347 | -0.073 | -0.011 |  | -0.240 |  |  | -0.065 | 0.064 |  |  | 11 | 1.59 | 0.034 |
| 5 | + | 0.342 | -0.077 |  | 0.027 | -0.252 |  |  |  |  |  |  | 9 | 1.80 | 0.031 |
| **Beech dry** | |  |  |  |  |  |  |  |  |  |  |  |  |  |  |
| 1 | + | 0.201 |  | 0.147 |  |  |  | + |  |  |  |  | 10 | 0 | 0.068 |
| 2 | + | 0.182 |  | 0.215 | 0.094 |  |  | + |  |  |  |  | 11 | 1.03 | 0.041 |
| 3 | + | 0.216 | -0.046 | 0.126 |  |  |  | + |  |  |  |  | 11 | 1.75 | 0.028 |
| 4 | + | 0.215 |  | 0.148 |  |  | -0.052 | + |  |  |  |  | 11 | 1.90 | 0.026 |
| 5 | + | 0.195 |  | 0.140 |  |  |  | + |  | 0.024 |  |  | 11 | 1.99 | 0.025 |
| **Beech wet** | |  |  |  |  |  |  |  |  |  |  |  |  |  |  |
| 1 | + | 0.847 | -0.715 | 0.092 | 0.001 | -0.345 | -0.306 | + |  |  |  |  | 13 | 0 | 0.333 |
| 2 | + | 0.845 | -0.705 | *0.017* |  | -0.580 |  |  | -0.135 | 0.082 |  |  | 11 | 0.57 | 0.250 |
| 3 | + | 0.845 | -0.699 | *0.038* | -0.110 | -0.421 |  | + | -0.082 |  |  |  | 13 | 1.17 | 0.186 |
| 4 | + | 0.802 | -0.703 |  | -0.053 | -0.401 |  | + |  |  |  | -0.097 | 13 | 2.68 | 0.087 |
| 5 | + | 0.839 | -0.730 |  | -0.006 | -0.559 |  |  |  |  | 0.115 | -0.150 | 11 | 2.85 | 0.080 |
| **Oak dry** | |  |  |  |  |  |  |  |  |  |  |  |  |  |  |
| 1 | + | 2.072 | 0.717 | 0.761 | -0.814 |  |  |  | -0.424 |  | 0.298 |  | 11 | 0 | 0.674 |
| 2 | + | 2.028 | 0.644 | 0.622 | -0.831 | -1.701 |  |  | -0.611 |  |  |  | 11 | 2.06 | 0.241 |
| 3 | + | 2.249 | 0.654 | 0.495 | -0.890 |  |  |  | -0.595 | -0.305 |  |  | 11 | 4.66 | 0.066 |
| 4 | + | 2.133 | 0.654 | 0.633 | -0.808 |  |  |  | -0.623 |  |  | 0.222 | 11 | 7.35 | 0.017 |
| 5 | + | 2.276 | 0.643 | 0.741 | -0.780 |  |  |  |  | -0.402 | 0.492 |  | 11 | 12.21 | 0.002 |
| **Oak wet** | | | |  |  |  |  |  |  |  |  |  |  |  |  |
| 1 | + | 0.331 | -0.382 | 0.453 | 0.342 |  |  |  |  |  | -0.528 | -0.292 | 11 | 0 | 0.995 |
| 2 | + | 0.503 | -0.4365 | 0.4293 | 0.1871 |  | -0.5426 |  |  |  | -0.6165 |  | 11 | 11.1 | 0.004 |
| 3 | + | 0.484 | -0.4344 | 0.4306 | 0.1983 | -0.5335 |  |  |  |  | -0.6086 |  | 11 | 14.5 | 0.001 |
| 4 | + | 0.435 | -0.403 | 0.4278 | 0.2286 |  |  |  |  | 0.1614 | -0.5665 |  | 11 | 16.2 | 0 |
| 5 | + | 0.489 | -0.4343 | 0.4351 | 0.1977 |  |  |  |  |  | -0.6107 |  | 10 | 22.1 | 0 |

**Table S3.** Correlation coefficients of mortality curves for the five studied species and the lag in years for the best correlation (in parentheses). Significant correlations in bold (*P* < 0.001).

|  | Oak | Spruce | Pine | Fir |
| --- | --- | --- | --- | --- |
| Beech | **0.34**  **(0)** | **0.54**  **(0)** | **0.57**  **(-2)** | **0.47**  **(0)** |
| Oak |  | 0.28  (0) | **0.46**  **(0)** | 0.32  (0) |
| Spruce |  |  | **0.53**  **(0)** | **0.62**  **(0)** |
| Pine |  |  |  | 0.20  (0) |

**Table S4.** Mean annual mortality rates (in % yr^-1^) for different time periods and species, and number of plots (N).

|  | **1970-2013** | | **1980-2013** | | **1990-2013** | | **2000-2013** | |
| --- | --- | --- | --- | --- | --- | --- | --- | --- |
|  | **Mean ± SE** | **N** | **Mean ± SE** | **N** | **Mean ± SE** | **N** | **Mean ± SE** | **N** |
| Pine | 1.5 ± 0.2 | 70 | 1.8 ± 0.27 | 50 | 2.3 ± 0.4 | 32 | 1.9 ± 1.44 | 4 |
| Spruce | 1.2 ± 0.13 | 58 | 1.2 ± 0.13 | 57 | 1.5 ± 0.26 | 32 | 1.5 ± 0.42 | 12 |
| Fir | 2.1 ± 0.22 | 37 | 1.8 ± 0.22 | 37 | 2.0 ± 0.29 | 31 | 1.9 ± 0.59 | 16 |
| Beech | 1.4 ± 0.14 | 100 | 1.4 ± 0.13 | 100 | 2.1 ± 0.34 | 66 | 1.3 ± 0.48 | 15 |
| Oak | 2.2 ± 0.19 | 78 | 2.1 ± 0.18 | 78 | 2.8 ± 0.27 | 49 | 2.4 ± 0.26 | 24 |
| Mean | 1.62 ± 0.09 | 276 | 1.67 ± 0.09 | 256 | 2.3 ± 0.18 | 165 | 1.92 ± 0.23 | 52 |

**Table S5** Summary of mortality rates reported in other studies from Europe listing studied region (CH: Switzerland, G: Germany, A: Austria, CZ: Czech Republic, Sw: Sweden, Ru: Russia, EU: Europe), time period, number of years spanning the studied time period, altitude of the study sites, plant group (angiosperms (A) or gymnosperms (G)), threshold of DBH, and the average annual mortality rate. “NA” indicates that information was not available, “~” indicates that mortality rates were visually estimated from figures in the publication.

| **Reference** | **Country** | **Time period** | **Total years** | **Altitude (m a.s.l.)** | **Plant group** | **Minimum DBH (cm)** | **Annual mortality rate (%)** |
| --- | --- | --- | --- | --- | --- | --- | --- |
|  |  |  |  |  |  |  | **Average** |
| This study | CH | 1900-2010 | 115 | 356-2119 | A,G | 5 | 1.5 |
| Braun (2016) | CH | ~1980-2011 | ~31 | 260-1870 | A,G | 16 | 0.28 |
| Rigling et al. (2013) | CH | 1983-2003 | 20 | <1000 | G | 12 | 0.1 |
| Rohner et al. (2012) | CH | 1956-2007 | 51 | 420-880 | A | 4 | 2.9 |
| Dobbertin et al. (2009) | CH | 1996-2005 | 9 | 330-1850 | A,G | NA | 0.2 |
| STMELF (2017) | G | 2011-2017 | 6 | NA | A,G | NA | 0.41 |
| Meining et al. (2017) | G | 1985-2017 | 32 | NA | A,G | NA | ~0.19 |
| Paar and Dammann (2017) | G | 1985-2017 | 32 | NA | A,G | NA | ~0.3 |
| Dammann and Paar (2017) | G | 1984-2017 | 33 | NA | A,G | NA | 0.1 |
| Pretzsch et al. (2014a) | G | 1960-2010 | 50 | 330-850 | A,G | NA | ~1.8 |
| Monserud and Sterba (1999) | A | 1986-1990 | 4 | NA | A,G | 10.4 | 1 |
| Janik et al. (2016) | CZ | 1974-2011 | 37 | 730-808 | A,G | 10 | 1.14 |
| Linder (1998) | Sw | 1922-1994 | 72 | 410 | G | NA | 0.45 |
| Hytteborn et al. (1991) | Sw | 1935-1989 | 54 | NA | G |  | 1.12 |
| Hytteborn et al. (2017) | Sw | 1912-2013 | 101 | 3-9 | A | 12 | 2 |
| Fraver et al. (2008) | Sw | 1986-2004 | 18 | 550 | A,G | 10 | 0.88 |
| Aakala et al. (2011) | Ru | 1980-1995 | 15 | 170-215 | G | 10 | 0.49 |
| Neumann et al. (2017) | EU | 2000-2012 | 12 | NA | A,G | NA | 0.5 |
| **Median** |  |  |  |  |  |  | **0.5** |

**Figure S1.** Locations of plots for the studied species and ecoregions.

**Figure S2.** Curves illustrating predicted response of annual mortality rates to ∆SWB_min_ and ∆Temp for different levels (0.25, 0.5 and 0.75% quantile) of basal area (BA) and mean DBH (mDBH) as derived from the GLMMs in **Table 3**. Response curves were calculated by holding all predictors in the model constant at their mean or, for BA and mDBH, at their respective quantile range, except for the selected displayed variable. Curves represent a mean response over the three DBH classes and four aspect levels (if significant in the model). The figures illustrate curves for dry and low altitude ecoregions per species.

**Figure S3.** Curves illustrating predicted response of annual mortality rates to ∆SWB_min_ and ∆Temp for different levels (0.25, 0.5 and 0.75% quantile) of basal area (BA) and mean DBH (mDBH) as derived from the GLMMs in **Table 3**. Response curves were calculated by holding all predictors in the model constant at their mean or, for BA and mDBH, at their respective quantile range, except for the selected displayed variable. Curves represent a mean response over the three DBH classes and four aspect levels (if significant in the model). The figure illustrates curves for wet and high-altitude ecoregions per species.
